# PCV2 infection induces the differentiation of Treg cells via the TGF-β/Smad3 pathway

**DOI:** 10.1128/mbio.01366-25

**Published:** 2025-07-31

**Authors:** Ruijiao Jiang, Qiuyan Huang, Ruiting Shen, Yongning Zhang, Lei Zhou, Xinna Ge, Jun Han, Xin Guo, Hanchun Yang

**Affiliations:** 1 College of Veterinary Medicine, China Agricultural University 630101 , Beijing, Beijing, China; University of Colorado Anschutz Medical Campus, Aurora, Colorado, USA

**Keywords:** PCV2, CD4^+^T, Treg, Foxp3, TGF-β, Smad3, immunosuppression

## Abstract

In recent years, porcine circovirus type 2 (PCV2) has become a significant pathogenic virus in the swine industry, causing huge economic losses globally. However, the impact of PCV2 on the differentiation of CD4^+^ T cells (Th1/Th2/Th17/Treg) remains unclear. In this study, we noticed that PCV2-infected piglets exhibited several clinical symptoms, including slow weight gain, diarrhea, and lethargy. In addition, we observed significant changes in the levels of IFN-γ, IL-4, and IL-17A during the early stages of infection, while the inhibitory cytokine IL-10 exhibited a significant increase in the later stages. Furthermore, the overall count of CD4^+^ T helper cells was significantly reduced, and the Th1/Th2/Th17/Treg immune balance was disrupted, with a shift towards Treg cells. What is more, we revealed that TGF-β, a cytokine that induces Treg cell differentiation, was highly expressed after PCV2 infection. This cytokine recruited and phosphorylated Smad3, which subsequently translocated into the nucleus to facilitate Foxp3 transcription. Besides, we also investigated the association between the changes in the intestinal microbiota caused by PCV2 infection and the immune balance of T cells. Overall, our findings enriched the mechanism of PCV2 promoting Treg cell differentiation and provided valuable insights for the prevention and treatment of immunosuppressive diseases.

**IMPORTANCE:**

Porcine circovirus type 2 (PCV2) infection can cause immunosuppression-related diseases in pigs. Currently, it is still recognized as an important infectious pathogen of the swine industry in the world. In this study, we discovered that PCV2 infection disrupted the Th1/Th2/Th17/Treg immune equilibrium, and the differentiation capacity of Treg cells increased significantly. Briefly, PCV2 infection promoted the secretion of cytokine TGF-β, recruited Smad3, and phosphorylated it. Subsequently, the phosphorylated Smad3 transmitted the signal from the cell membrane to the nucleus and bound to the enhancer of Foxp3, thereby enhancing the transcription level of Foxp3 and facilitating the differentiation of Treg cells. This study enriches the pathogenic mechanism of PCV2 persistent infection and provided a theoretical basis for the prevention and control of immunosuppressive diseases.

## INTRODUCTION

Porcine circovirus mainly includes PCV1, PCV2, PCV3, and PCV4 genotypes, among which PCV2 is the most pathogenic and prevalent genotype at present (1, 2). PCV2, a non-enveloped single-stranded DNA virus, belongs to the genus *Circovirus* in the family *Circoviridae*. The genome size of PCV2 is approximately 1.76 kb, which contains 11 open reading frames (ORFs) (3–5). Due to the overlap of ORFs, the virus can maximize the use of its limited genetic material to carry a wealth of genetic information. At present, PCV2 has been causing significant economic losses to the pig industry (6, 7). This “small” but “smart” virus not only has a high mutation rate to provide a theoretical possibility for its evolution and adaptation to the environment, but also, more importantly, it exhibits a variety of means to elude the host’s antiviral defenses and induce immune suppression (8–10). As a result, more and more clinical cases reported that PCV2-infected pigs tend to present subclinical symptoms (11), viral mixed infection (12, 13), and secondary bacterial infection (14, 15).

Current evidence demonstrates that porcine circovirus type 2 (PCV2) primarily targets immune organs, inducing significant lymphocyte depletion (16, 17). Furthermore, PCV2 infection leads to quantitative reductions in multiple immune cell populations, including dendritic cells (DCs), B cells, natural killer (NK) cells, γδ T cells, and both CD4^+^ and CD8^+^ T cells. This widespread immunocyte depletion compromises the host’s capacity to eliminate existing pathogens and impairs defensive responses against secondary infections. Among these, CD4^+^ T cells are a key component of the immune system (18, 19), and the changes in their different subtypes will directly or indirectly affect the immune state and inflammatory processes of the host. Recent studies have demonstrated that PCV2 infection promotes the differentiation of regulatory T cells (Treg cells, a subset of CD4^+^ T cells) (20, 21), with TGF-β playing a pivotal regulatory role in this process (22, 23). However, the precise impact of PCV2 infection on the immunological balance of CD4^+^ T cell subsets (including Th1, Th2, Th17, and Treg cells) remains unclear. Furthermore, the molecular mechanisms through which TGF-β activates downstream signaling pathways have yet to be elucidated. Therefore, this study aims to investigate the impact of PCV2 infection on the Th1/Th2/Th17/Treg immune balance and elucidate the underlying mechanisms governing Treg cell differentiation. The findings will provide a theoretical foundation for PCV2 prevention and control while offering new perspectives for vaccine design and pharmaceutical development.

## MATERIALS AND METHODS

### Animals, cells, and virus

Eight 35-day-old Sanyuan piglets were a gift from the Harbin Guosheng Biotechnology Co., Ltd. All piglets used in this study were derived by cesarean section from the same SPF sow and were artificially fed after birth. All animals were free of PCV2, porcine reproductive and respiratory syndrome virus (PRRSV), classical swine fever virus (CSFV), and pseudorabies virus (PRV) and were seronegative for PCV2, PRRSV, and CSFV.

PK-15, IPEC-J2, and HEK-293T cells were cultured in Dulbecco’s Modified Eagle Medium (DMEM; 12800017, GIBCO) supplemented with 10% fetal bovine serum (FBS, 10099141C, GIBCO) and 1% penicillin-streptomycin solution (C0222, Beyotime). 3D4/21 cells were cultured in RPMI-1640 supplemented with 10% FBS and 1% penicillin-streptomycin. Peripheral blood mononuclear cells (PBMCs) were cultured in RPMI-1640 supplemented with 10% FBS and 1% penicillin-streptomycin (RPMI-1640 complete medium). T cells were cultured in RPMI-1640 supplemented with 10% FBS, 1% penicillin-streptomycin, 5 µg/mL ConA (C2010, Sigma-Aldrich), 1× MEM non-essential amino acid (M7145, Sigma-Aldrich), and 1 mM sodium pyruvate (S8636, Sigma-Aldrich).

The PCV2d strain (GenBank: OR232686) preserved in our laboratory was propagated on PK-15 cells, and the virus titer was determined to be 10^6^ TCID_50_/mL.

### Chemicals and antibodies

PMA + ionomycin (2030421) was purchased from Dakewe Biotech Co., Ltd. Brefeldin A (50502ES03) was purchased from Yeasen Biotechnology (Shanghai) Co., Ltd. Cell lysis buffer for Western and IP (P0013) and PMSF (ST505) were purchased from Beyotime. SIS3, a specific Smad3 phosphorylation inhibitor (HY-13013), and SB-431542, a TGF-β receptor inhibitor (HY-10431), were purchased from MedChemExpress.

PE mouse anti-pig CD3ε (561485), PerCP-CyTM5.5 mouse anti-pig CD4α (561474), PE mouse anti-human IL-17A (560436), and Alexa Fluor 647 mouse anti-pig IFN-γ (561480) were purchased from BD Pharmingen. Alexa Flour 647 mouse anti-pig CD25 (MCA1736A647) was purchased from Bio-Rad. Gata-3 monoclonal antibody (TWAJ) PE (12-9966-42) and FOXP3 monoclonal antibody (FJK-16s) PE (12-5773-82) were purchased from Invitrogen. Beta actin monoclonal antibody (66009-1-Ig), FOXP3 polyclonal antibody (22229-a-AP), and Smad3 monoclonal antibody (66516-1-Ig) were purchased from Proteintech. Phospho-Smad3 (Ser423 + Ser425) antibody (AF8315) was purchased from Affinity Biosciences.

### Animal experimental design

Eight piglets were randomly divided into two groups: three in the control group and five in the challenge group. The piglets in the challenge group were inoculated with 3 mL PCV2 virus in each nasal cavity (i.e., the challenge dose was 6 × 10^6^ TCID_50_) at 35 days of age (0 days post-inoculation, dpi), and the control group was inoculated with the same volume of PBS. Piglets were raised in isolation and fed 500 g per day of a balanced, pelleted, complete feed ration free of antivirus and antibiotics. Following PCV2 inoculation, the live weight of the pigs was measured every seven days, and the average daily weight gain (ADWG) was analyzed for five consecutive weeks. Additionally, clinical symptoms in pigs were monitored and scored daily, including but not limited to body temperature, anorexia, consciousness status, and respiratory signs. Detailed scoring criteria were provided in Table S1.

### PCV2 DNA quantification

Genomic DNA was extracted from piglet serum samples collected at 0, 7, 14, 21, 28, and 35 dpi, as well as post-euthanasia tissue samples, using the TIANamp Genomic DNA Kit (DP304-03, TIANGEN). In order to quantify PCV2 DNA in serum or tissue samples, we established a real-time PCR to achieve that. In a nutshell, the PCV2d fragment was amplified, linked to the pET-28a plasmid, converted to DH5ɑ, and identified by PCR. The correct plasmid, pET-28a-Rep, was first quantified to 1 × 10^10^ copies/μL, followed by a 10-fold gradient dilution as a DNA template. All the RT-qPCR reactions were carried out by the Bio-Rad CFX96 Touch Real-Time PCR system, and the reaction system was as follows: 2× Taq Pro Universal SYBR qPCR Master Mix (Q712, Vazyme) was 10 µL, upstream and downstream primers were 0.5 µL, DNA was 2 µL, and RNase-free water was 7 µL. The qPCR conditions were as follows: initial denaturation for 5 min at 95°C, followed by 40 cycles of 95°C for 30 s and 60°C for 30 s. A melting curve analysis was performed to ensure the specificity of the products. Finally, a standard curve was drawn to detect the PCV2 DNA copies in unknown samples. Primers used in this study have been listed in Table S2.

### Serological test

Serum samples were collected for the detection of PCV2-specific antibodies at 0, 7, 14, 21, 28, and 35 dpi. All samples were detected using the PCV2-dCap-ELISA Ab kit (JN60315, Jinnuo Baitai Biotechnology) according to the manufacturer’s protocols.

### Histopathology and immunohistochemistry (IHC)

All pigs were sacrificed at the end of the experiment (35 dpi). Necropsy was performed for the characterization of gross lesions, and samples of the heart, liver, spleen, lung, kidney, tonsil, and lymph nodes were collected for IHC examinations. In short, all samples were fixed in 4% paraformaldehyde, embedded in paraffin wax, and sectioned at 3 to ~5 µm. The sections were mounted on SuperFrost Plus slides (Mensel-Gläser, Braunschweig, Germany) for IHC.

### RNA extraction and RT-qPCR assay

Total RNA from PBMCs was extracted using the TRIzol reagent (BioMed) according to the manufacturer’s recommendations. Then RNA (200 ng) was reverse transcribed with the HiScript III All-in-one RT SuperMix Perfect for qPCR Kit (R333,Vazyme) following the manufacturer’s instructions. The cDNA samples were quantified by Taq Pro Universal SYBR qPCR Master Mix. The reaction system and the qPCR conditions were the same as method 2.4. Primers used in this study have been listed in Table S2.

### Flow cytometry

Anticoagulant blood was collected at 0, 7, 14, 21, 28, and 35 dpi, and PBMCs were isolated with the pig peripheral blood lymphocyte separation solution kit (LTS1110, Tianjin Haoyang). PBMCs were prepared into a single-cell suspension using RPMI-1640 complete medium, and the concentration was adjusted to about 2 × 10^7^ cells/mL. Three 100 µL PBMCs (2 × 10^6^ cells) were labeled as A, B, and C tubes for Th1 (CD4^+^IFN-γ^+^)+Th2 (CD4^+^GATA3^+^), Th17 (CD4^+^IL-17A^+^), and Treg (CD4^+^CD25^+^Foxp3^+^) detection, respectively. Three tubes of PBMCs were placed in 12-well plates and cultured in complete RPMI-1640 medium. In order to stimulate cells and limit the release of cytokines, PBMCs were treated with PMA + ionomycin and brefeldin A at 37°C in a 5% CO_2_ incubator for 5 h. Then, the single-cell suspension of each well was centrifuged, and the supernatant was discarded to collect cells.

A Foxp3/Transcription Factor Staining Buffer Kit (IC001, Multi Sciences) was used to mark cells according to the manufacturer’s protocols. In brief, the collected cells were resuspended with 50 µL PBS and incubated with CD3ε antibody/CD4α antibody/CD25 antibody as required on ice for 30 min to label surface molecules. After the cells were fixed and broken, intracellular antibodies were added to different groups as required and incubated at room temperature for 30 min away from light. After washing, the labeled cells were resuspended with PBS containing 1% BSA, and the samples can be detected on the flow cytometer (BD Pharmingen). All data were analyzed using the FlowJo software.

### Cytokine detection

Serum samples were collected at 0, 7, 14, 21, 28, and 35 dpi. Commercial pig sandwich ELISA kits (YJ771030, YJ771047, YJ771038, YJ771034, Yuanju Bio) were used to test the levels of IFN-γ, IL-4, IL-17A, and IL-10 according to the manufacturer’s instructions. The cytokine secretion levels of different T cell subtypes were preliminarily characterized. Serum samples collected at 35 dpi were analyzed for IL-2 and TGF-β levels using commercial ELISA kits (ml002305, ml002363, Mlbio). To analyze cytokine secretion levels *in vitro*, cell culture supernatants were collected 48 hours post-infection (hpi) and were quantitatively assessed.

### SDS-PAGE and Western blot assay

Cells were washed with PBS three times and lysed in cell lysis buffer with the protease inhibitor PMSF on ice for 1 h. The lysate was centrifuged at 12,000 rpm for 30 min, isolated by SDS-PAGE electrophoresis, and then transferred to a PVDF membrane. The membrane was blocked in PBS with 5% skimmed milk at room temperature for 2 h, followed by incubation with the primary antibody at 4℃ overnight, and then incubated with the HRP-conjugated secondary antibody for 1 h at room temperature. Subsequently, the protein band was obtained by using an Enlight reagent (17100, Engreen) via a bioanalytical imaging system.

### Isolation of T cells

T cells were isolated using an established nylon wool column method (24, 25). Briefly, 1.5 g of nylon wool was packed into a 20 mL syringe (filling approximately 1/3–1/2 of the column volume). After removing the needle and connecting a three-way stopcock, the column was autoclaved for sterilization. Then, the column was pre-washed with pre-warmed RPMI 1640 medium and equilibrated with 3 mL of RPMI 1640 containing 2% FBS at 37°C for 1 h. After draining, 4 mL of PBMC suspension (~3 × 10^8^ cells) was loaded onto the column and incubated at 37°C for 1 h. The column was then eluted at a flow rate of 1 drop/sec while continuously supplementing with RPMI 1640 until the effluent became clear. The eluted cells were collected by centrifugation, and T cell purity was verified by flow cytometry before cryopreservation.

### MTT assay

T cells were isolated from PBMCs for the MTT assay. In brief, T cells were resuspended in RPMI-1640 medium containing 10% FBS, 1% penicillin-streptomycin, and 5 µg/mL ConA (used to stimulate T cell proliferation). A 100 µL suspension was inoculated into a 96-well microcell culture plate on the bottom of the plate with approximately 1 × 10^5^ cells per well. Samples cultured in an incubator at 37°C with 5% CO_2_ for 72 h were used to detect proliferation according to the MTT cell proliferation and cytotoxicity detection kit.

### Plasmids and transfection

To validate the regulatory effect of Smad3 on Foxp3 transcription, we performed a comparative sequence analysis between porcine and human Foxp3, based on the findings by Tone et al. (26), and identified potential Smad3-binding motifs. Subsequently, we generated hexameric repeats of the target sequence and cloned them into the pGL3-promoter reporter plasmid (pGL3-promoter-Foxp3). And *Smad3* from PK-15 genomic DNA was amplified and cloned into pCAGGS-HA. Successfully constructed plasmids were transfected with Lipofectamine 3000 (L3000015, Invitrogen) into HEK-293T cells that had already grown a full monolayer. Then, the plasmid expression was assessed according to Method 2.10 to determine whether the plasmids were successfully expressed.

### Dual-luciferase reporter assay

In order to verify whether Smad3 can bind pig Foxp3 enhancer and promote Foxp3 transcription, dual-luciferase reporter assay (E1910, Promega) was performed according to the instructions.

### Establishment of T cell–host cell co-culture system

To investigate whether PCV2 infection of host cells could promote cytokine secretion and subsequent Treg cell differentiation, an *in vitro* T cell–host cell co-culture system was established. Briefly, 1.5 mL serum-free RPMI-1640 medium was added to the lower chamber of a 12-well Transwell plate, while 0.5 mL of the same medium was added to the upper chamber. After 2 h pre-equilibration at 37°C, the medium was completely removed. Host cells (PK-15, IPEC-J2, and 3D4/21) in logarithmic growth phase were then seeded in the lower chamber. When reaching 90% confluence, cells were infected with PCV2 at MOI = 0.1 for 1 h at 37°C, followed by PBS washing and medium replacement. Pre-stimulated T cells (12–24 h) were adjusted to 2 × 10^6^ cells/mL and added to the upper chamber. The co-culture system was maintained for 72 h at 37°C with 5% CO_2_ before subsequent supernatant or cell collection for further analysis.

### Statistical analysis

All the experiments were performed with at least three independent replicates. GraphPad 7.0 (GraphPad Software, San Diego, CA, USA) was used to analyze our data, and differences were analyzed using two-way ANOVA. A *P*-value < 0.05 was considered significant, and a *P*-value < 0.01 was considered highly significant. Each value was the mean ± standard deviation (SD) of three replicates.

## RESULTS

### PCV2 infection induced growth retardation in piglets

**Fig 1:**
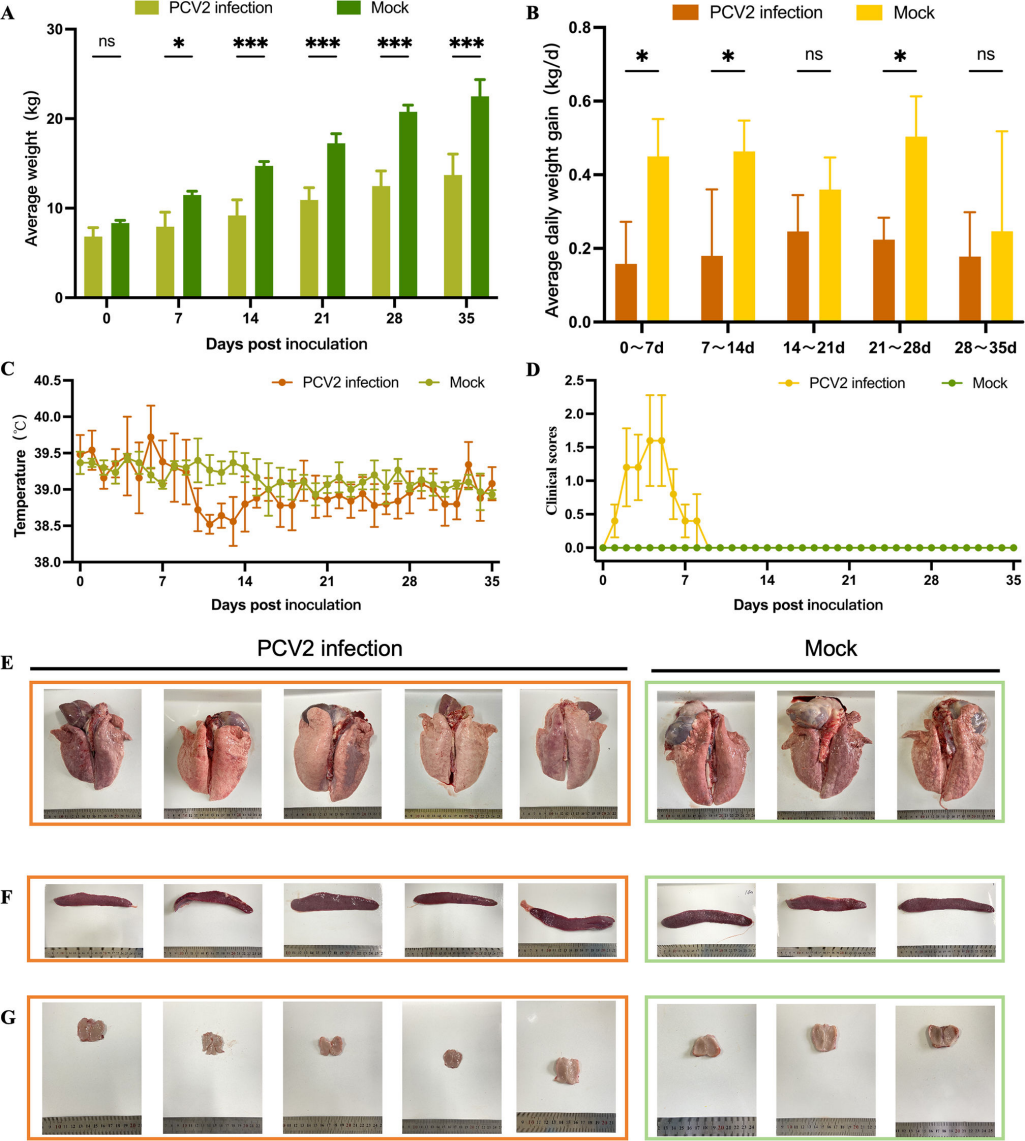
(**A**) Clinical indication analysis and pathological observation. The average weight of piglets. Body weights of all experimental animals were recorded weekly. The mean body weight (±SD) was calculated for each group (mock: *n* = 3; PCV2 infection: *n* = 5). (**B**) The average daily weight gain of piglets. The average daily gain was calculated based on weekly weight measurements. (**C**) Temperature monitoring. The rectal temperature of piglets was measured each morning before feeding. (**D**) Clinical score. Clinical scores of all piglets were recorded following daily morning observations prior to feeding. All piglets were euthanized at 35 dpi, followed by gross pathological examination of the lungs (**E**), spleen (**F**), and tonsils (**G**). The orange boxes represent the challenge group, and the green ones represent the control group.

In this study, SPF piglets were challenged with the currently globally prevalent PCV2d strain to systematically evaluate its pathogenic characteristics. The results demonstrated that while no mortality was observed in the PCV2 infection group, significant growth retardation occurred, with reduced average body weight (Fig. 1A) and daily weight gain (Fig. 1B) compared to the control group. Generally, PCV2-infected piglets exhibited mild temperature fluctuations (Fig. 1C) and clinical manifestations including diarrhea, lethargy, and anorexia, leading to significantly elevated clinical scores during the early infection phase (Fig. 1D). In addition, gross pathological examination revealed no significant lesions in target organs, such as lungs, spleen, and tonsil (Fig. 1E through G).

### PCV2 exhibited stronger tissue tropism for lymphoid organs

**Fig 2:**
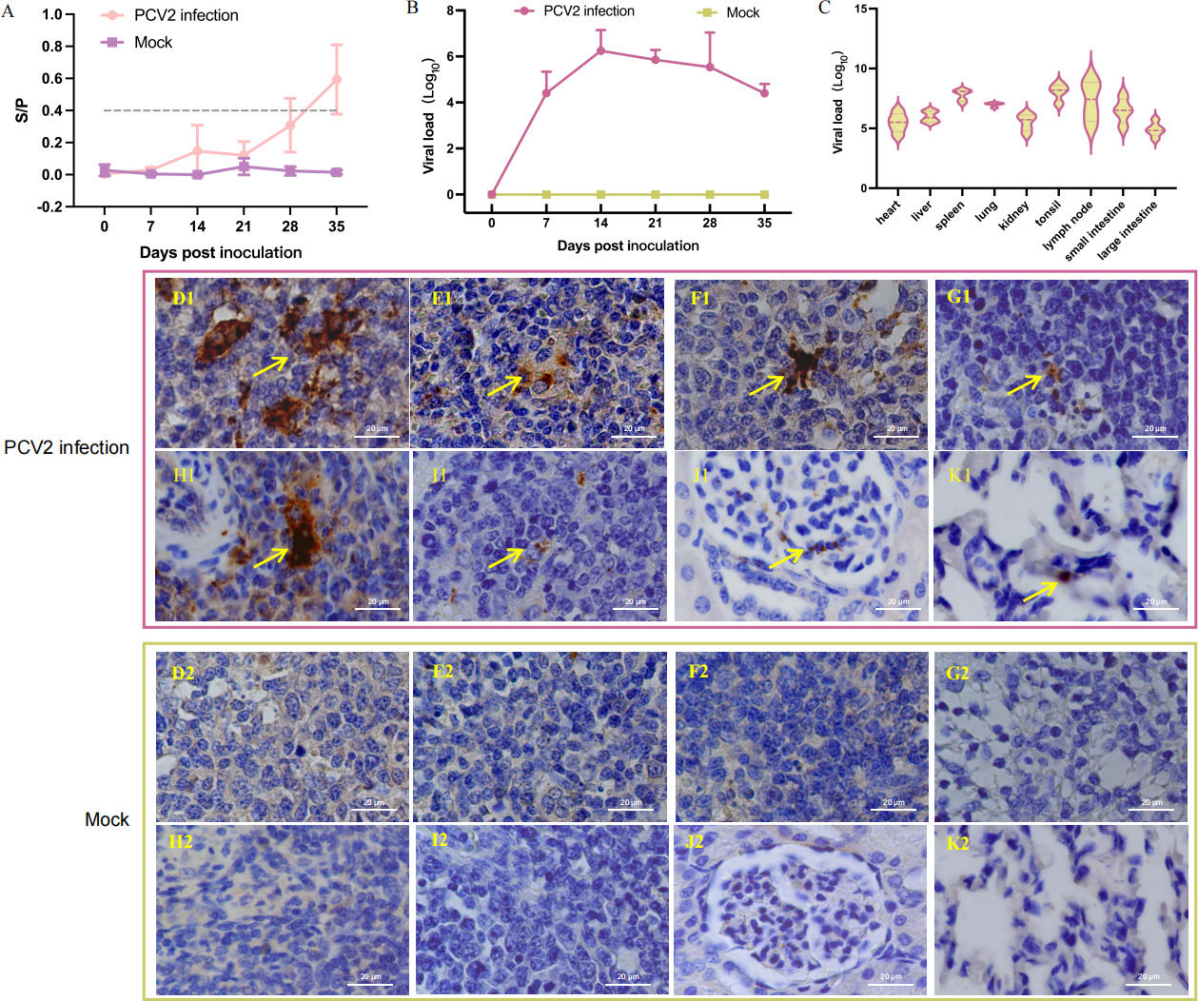
Detection of PCV2 antigen and antibody and immunohistochemical analysis. (**A**) Antibody level monitoring. Serum PCV2 antibody levels were measured by ELISA at different time points after PCV2 challenge. (**B**) Detection of PCV2 DNA copy number in peripheral blood. Serum PCV2 viral load was quantified using an established qPCR assay. (**C**) Viral load analysis of PCV2 in tissues. All piglets were euthanized at 35 dpi. Tissue samples (1 mg each) were collected for DNA extraction, followed by qPCR analysis of PCV2 viral load in different tissues. (**D–K**) Immunohistochemical detection. At 35 dpi, all piglets were euthanized. Tissue samples, including mandibular, mesenteric, inguinal, and hilar lymph nodes, spleens, tonsils, kidneys, and lungs, were collected for PCV2 immunohistochemical analysis, respectively. The numbers 1 and 2 successively represent piglets in the challenge group and the control group.

To elucidate the infection kinetics of PCV2 in piglets, we initially monitored PCV2-specific antibody levels in serum samples collected at different time points post-infection using ELISA. As shown in Fig. 2A, the antibody level of piglets began to increase at 28 dpi, and all piglets were PCV2 seropositive at 35 dpi. Subsequently, a qPCR assay was established using the constructed plasmid pET-28a-Rep to precisely evaluate viral copy numbers in serum samples. The PCV2 viral load in each tissue sample was calculated according to the standard curve *Y* = −3.502(lg*X*) + 41.564 (Fig. S1). As shown in Fig. 2B, viremia was first detected in serum at seven dpi with PCV2, peaked at 14 dpi, and subsequently exhibited a gradual decline.

To monitor the PCV2 viral load in different tissues of infected piglets, all piglets were sacrificed at 35 dpi. The copy number of PCV2 in different tissues was determined by the established qPCR detection method. As shown in Fig. 2C, the histotropism of PCV2 is very extensive and can be detected in almost all organs, with a relatively high viral load in the spleen, lymph nodes, and tonsils. Furthermore, we performed IHC analysis on these organs of PCV2 infection. The results showed that a large number of PCV2 pathogens were detected in the lymph nodes of five pigs in the challenge group, especially in mandibular lymph nodes (Fig. 2D), followed by mesenteric lymph nodes (Fig. 2E), inguinal lymph nodes (Fig. 2F), and hilar lymph nodes (Fig. 2G). Additionally, a large number of PCV2 pathogens were also detected in the spleen (Fig. 2H), while very little was detected in the tonsils, kidneys, or lungs (Fig. 2I through K).

### PCV2 infection disrupted the Th1/Th2/Th17/Treg cell balance and promoted Treg cell differentiation

Following PCV2 infection in piglets, we first examined T cell proliferative capacity and observed significant suppression in the PCV2 infection group (Fig. S2A). Early-stage infection showed marked reduction in CD4^+^ T cell counts, with gradual recovery thereafter (Fig. S2B). To further investigate CD4^+^ T cell subset dynamics, we isolated PBMCs at various post-infection time points and quantified distinct subsets by qPCR analysis of T cell-specific transcription factors (Fig. S3). We found that at seven dpi, the T-bet transcription factor, which characterizes Th1 cells, was significantly reduced. Of particular note, at 28 dpi or 35 dpi, the Foxp3 transcription factor, which characterizes Treg cells, showed significantly higher levels in the challenge group. This was one of the few T cell types to increase after PCV2 infection.

**Fig 3:**
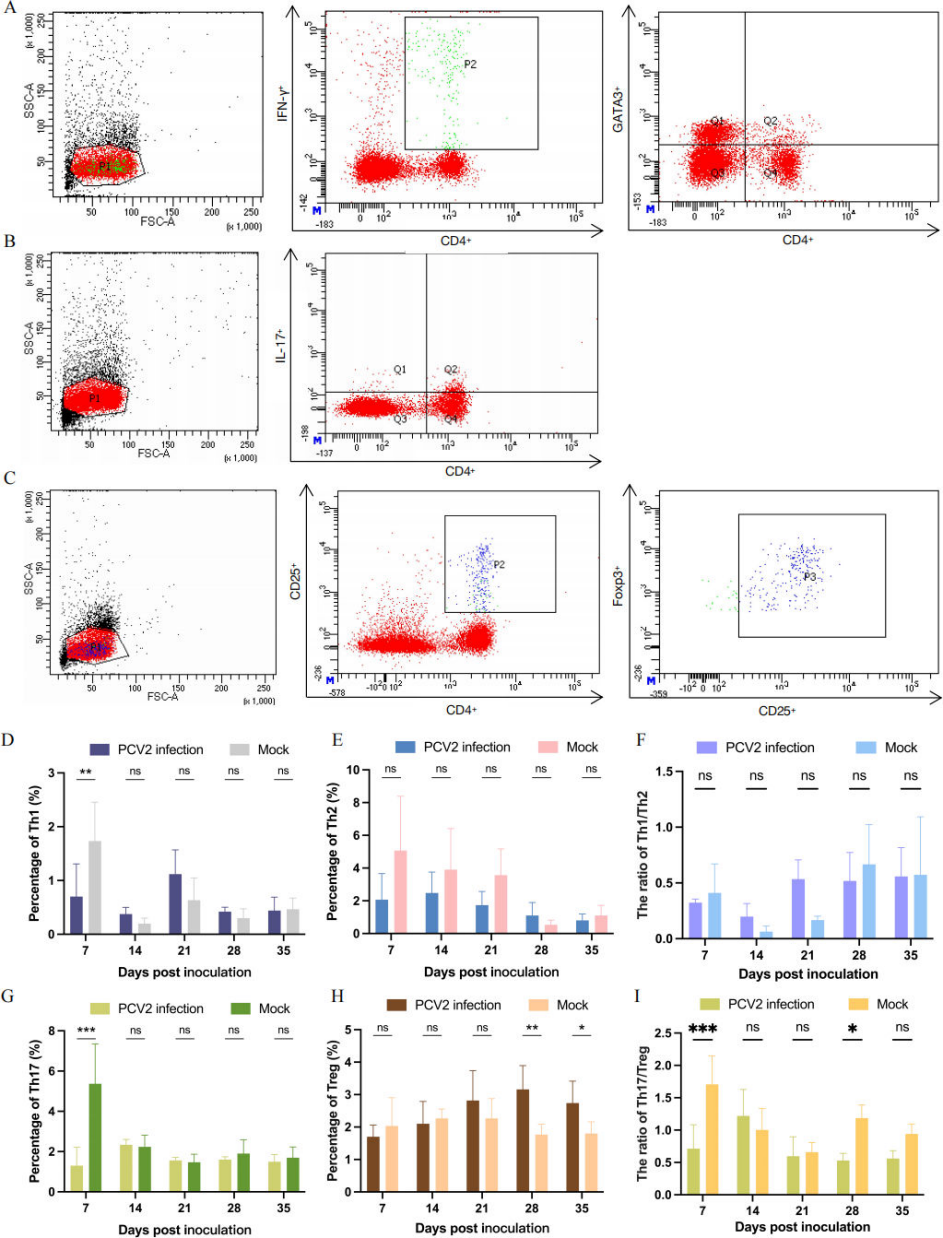
(**A**) CD4^+^ T cell immune balance detection. Th1 and Th2 cells were detected simultaneously by flow cytometry. Th1 cells were labeled with CD4^+^IFN-γ^+^ (PerCP-CyTM5.5 mouse anti-pig CD4α, Alexa Fluor 647 mouse anti-pig IFN-γ), and Th2 cells were labeled with CD4^+^GATA3^+^ (PerCP-CyTM5.5 mouse anti-pig CD4α, Gata-3 monoclonal antibody [TWAJ], PE). (**B**) Th17 cells were detected by flow cytometry. They were labeled with CD4^+^IL-17A^+^(PerCP-CyTM5.5 mouse anti-pig CD4α, PE mouse anti-human IL-17A). (**C**) Treg cells were detected by flow cytometry. They were labeled with CD4^+^CD25^+^Foxp3^+^ (PerCP-CyTM5.5 mouse anti-pig CD4α, Alexa Fluor 647 mouse anti-pig CD25, FOXP3 monoclonal antibody (FJK-16s), PE). Dynamic changes in Th1 (**D**) and Th2 (**E**) cell frequencies were quantitatively analyzed at various time points following PCV2 infection. (**F**) Th1/Th2 immune balance analysis. The frequencies of Th17 (**G**) and Treg (**H**) cells in piglets were measured at different time points during PCV2 infection. (**I**) Th17/Treg immune balance analysis.

To accurately determine the immune balance between Th1/Th2 and Th17/Treg cell subclasses, flow cytometry was used to detect the frequency of different T cell subclasses (Fig. 3A through C). Through statistics, we noted that Th1 cells decreased only at seven dpi, and the total number did not fluctuate significantly throughout the subsequent experimental phase (Fig. 3D). During PCV2 infection, Th2 cell populations remained relatively stable (Fig. 3E). Analysis of the Th1/Th2 ratio revealed a balanced cellular and humoral immune response during the infection process (Fig. 3F). What is more, Th17 cells decreased significantly at seven dpi and then stabilized (Fig. 3G). The most noteworthy finding was the significantly higher presence of Treg cells compared to the control group at 28 dpi and 35 dpi (Fig. 3H). The results were consistent with the qPCR detection and represented the only increasing T cell subtype among the four that showed an increase. By analyzing the Th17/Treg ratio, we discovered that both at seven dpi and 28 dpi, the immune balance of Th17/Treg cells was in favor of Treg (Fig. 3I). Overall, the decrease in Th17 cells at seven dpi and the increase of Treg cells at 28 dpi were the main factors contributing to this phenomenon.

Given PCV2’s strong tropism for lymphoid organs, total RNA and proteins were extracted from the spleen and lymph nodes to quantify Foxp3 expression levels. Results showed that PCV2-infected piglets exhibited significantly higher Foxp3 mRNA and protein expression levels in the spleen compared to controls. Although Foxp3 expression was elevated in lymph nodes, the increases were not statistically significant (Fig. S4).

### PCV2 infection disrupted host cytokine secretion profiles

**Fig 4:**
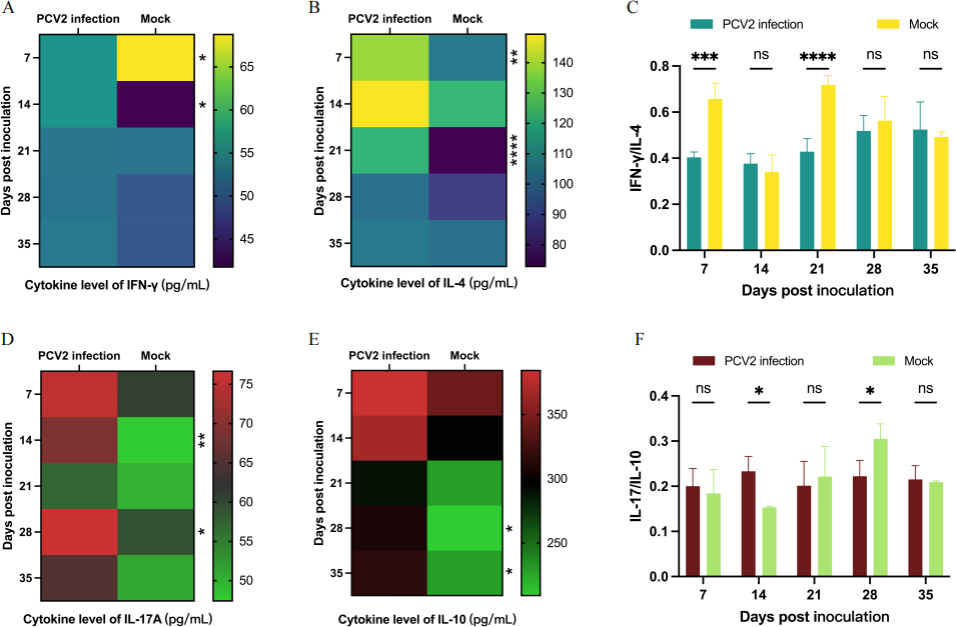
Detection of cytokine secretion levels. (**A and B**) Serum levels of the cytokines IFN-γ and IL-4 were detected by ELISA, respectively. Serum samples were collected from piglets at multiple time points, and cytokine levels were quantified by ELISA. (**C**) The IFN-γ/IL-4 ratio analysis. The IFN-γ/IL-4 ratio was calculated by dividing the IFN-γ concentration by the corresponding IL-4 level in each serum sample. (**D and E**) Serum levels of the cytokines IL-17A and IL-10 were detected by ELISA, respectively. (**F**) The IFN-γ/IL-4 ratio analysis.

Given that distinct T cell subsets secrete characteristic cytokines, we employed ELISA to quantify serum levels of IFN-γ (Th1), IL-4 (Th2), IL-17A (Th17), and IL-10 (Treg) in PCV2-infected piglets, thereby evaluating viral impact on T cell immune homeostasis. Specifically, at seven dpi, IFN-γ levels were significantly reduced in the stimulated group, whereas an opposite trend was observed one week later (Fig. 4A). Throughout the experimental period, the challenged group maintained higher IL-4 levels compared to controls, peaking at 21 dpi (Fig. 4B). The IFN-γ/IL-4 ratio analysis revealed a significant decrease during early PCV2 infection (Fig. 4C), demonstrating a Th2-polarized immune response. Notably, PCV2 infection induced early IL-17A upregulation (Fig. 4D) followed by progressive IL-10 elevation (Fig. 4E). The results of the IL-17A/IL-10 ratio showed that the piglets in the challenge group exhibited a trend of first increasing and then decreasing, suggesting the eventual establishment of Treg cell-mediated immunomodulation (Fig. 4F). Collectively, our findings demonstrate distinct temporal patterns: IFN-γ, IL-4, and IL-17A showed prominent variations during early vaccination, while IL-10 dynamics were primarily altered in later stages.

### PCV2 infection induced Treg cell differentiation through the TGF-β/Smad3 pathway

**Fig 5:**
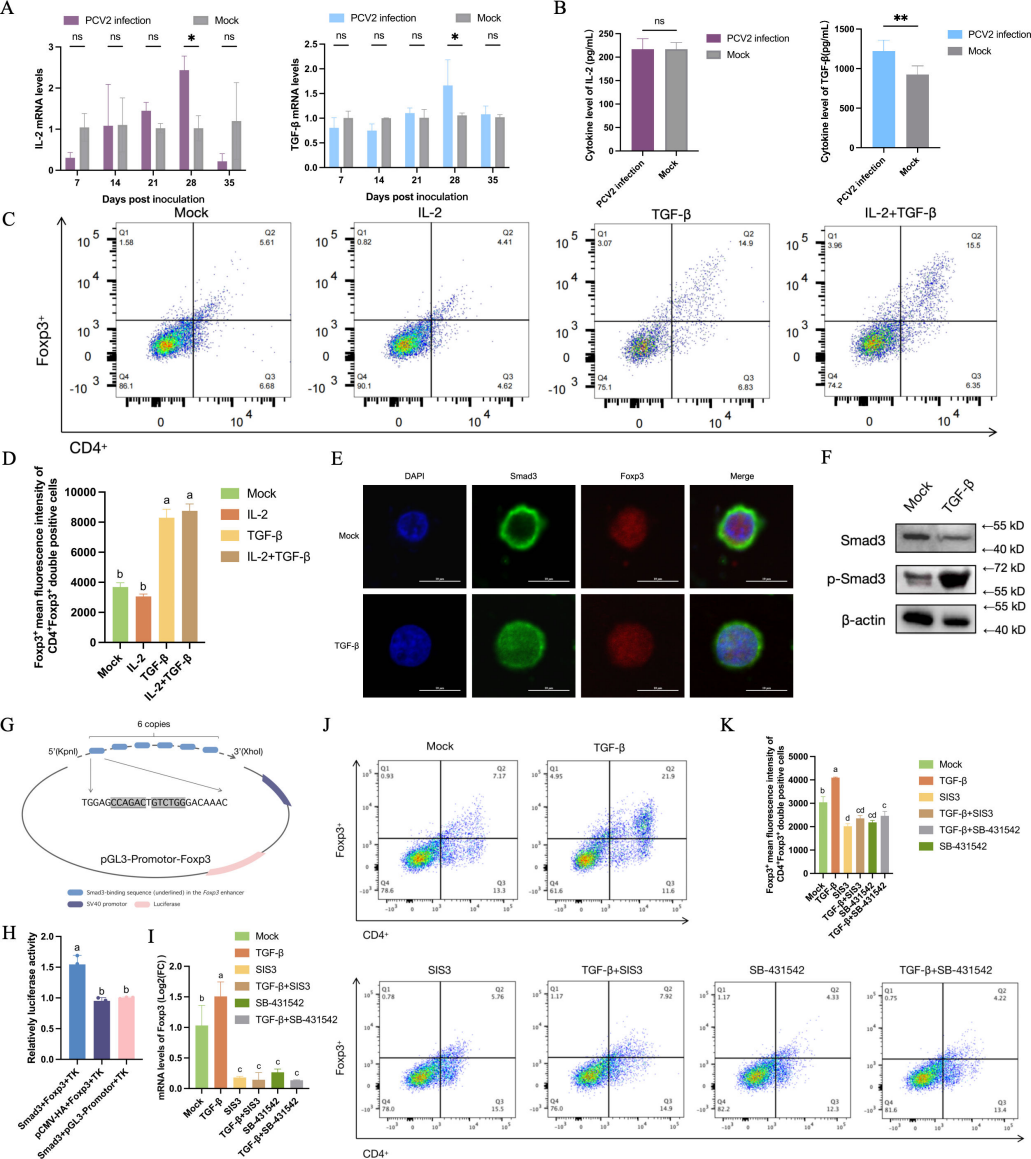
(**A**) PCV2 infection induced Treg cell differentiation through the TGF-β/Smad3 pathway. Detection of the mRNA levels of IL-2 and TGF-β. PBMCs were isolated from peripheral blood at multiple time points post-challenge, and the mRNA expression levels were quantified by qPCR. (**B**) Detection of the expression levels of IL-2 and TGF-β. All animals were euthanized at 35 dpi, and cytokine levels were measured by ELISA. (**C**) The frequency of Treg cells. T cells were stimulated with various cytokines and analyzed for Treg cell frequency by flow cytometry. Unless otherwise specified, T cell stimulations lasted five days *in vitro*. (**D**) The MFI of Foxp3 in CD4^+^Foxp3^+^ double-positive cells. Foxp3 expression in Treg cells was quantified by calculating the arithmetic MFI using flow cytometry. (**E**) The subcellular localization of Smad3 in Treg cells. (**F**) Detection of phosphorylation level of Smad3. Total proteins were extracted from T cells and analyzed by Western blot for target protein expression. (**G**) pGL3-promoter-Foxp3 schematic diagram. (**H**) The double luciferase assay. The assay was used to investigate the effect of Smad3 on the activity of the Foxp3 enhancer. (**I**) Quantification of Foxp3 mRNA expression levels. (**J**) The frequency of Treg cells. (**K**) The MFI of Foxp3 in CD4^+^Foxp3^+^ double-positive cells.

We speculated that PCV2 infection induced Treg cell differentiation in two ways: one is that PCV2 could infect T cells and promote differentiation of Treg cells by inducing Foxp3 transcription levels, and the other is that PCV2-infected cells secreted cytokines IL-2 or TGF-β to promote Treg cell differentiation. Firstly, T cells were infected with PCV2 *in vitro*, followed by analysis of Foxp3 expression levels and Treg cell frequency. However, no significant differences were observed in Foxp3 mRNA levels, protein expression, or Treg cell numbers between infected and control groups (Fig. S5). These findings demonstrate that PCV2 infection does not directly induce Treg cell differentiation. Therefore, we further detected cytokines IL-2 and TGF-β, and the results showed that the TGF-β mRNA and protein levels of cytokines in the challenge group were significantly higher than those in the control group (Fig. 5A and B). Furthermore, IL-2 or TGF-β was added to T cell medium, and the amount of Treg cells was analyzed by flow cytometry. We found that TGF-β is the key cytokine inducing Treg cell differentiation (Fig. 5C), which can be demonstrated by both the number of Treg-positive cells and their mean fluorescence intensity (MFI) (Fig. 5D).

Smad3 is one of the crucial molecules for transmitting TGF-β signals into the nucleus. We observed that in T cells treated with TGF-β, Smad3 translocated ectopically into the nucleus (Fig. 5E; Fig. S6A) and underwent phosphorylation modification (Fig. 5F; Fig. S6B). However, treatment with the Smad3 inhibitor SIS3 or TGF-β receptor antagonist SB-431542 effectively suppressed Smad3 phosphorylation (Fig. S6C), confirming TGF-β/Smad3 pathway dependency. To investigate the molecular mechanism of Smad3-mediated downstream activation, we constructed a pGL3-promoter-Foxp3 plasmid containing six tandem copies of putative Smad3-interacting fragments (Fig. 5G). In addition, a pCAGGS-HA-Smad3 expression plasmid was also constructed *in vitro* (Fig. S7). The dual-luciferase reporter assay indicated that Smad3 indeed enhanced the activity of the Foxp3 enhancer and promoted its transcription (Fig. 5H). SIS3 or SB-431542 was used to inhibit the phosphorylation of Smad3 or block the TGF-β receptor, and it was discovered that both were capable of suppressing the transcription of Foxp3 (Fig. 5I) and the differentiation of Treg cells (Fig. 5J and K).

### PCV2 infection promoted Treg cell differentiation through co-culture with host cells and T cells

**Fig 6:**
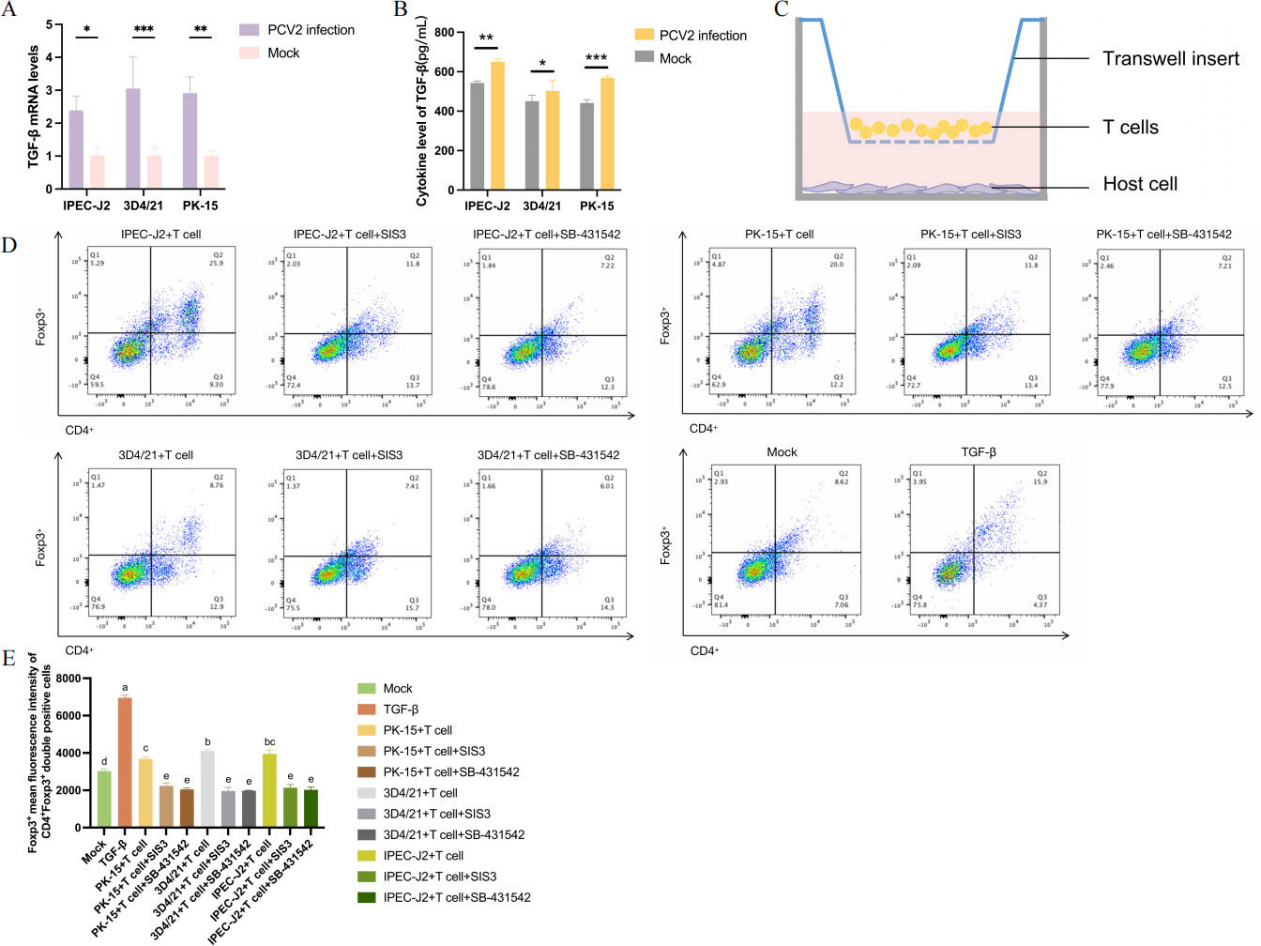
PCV2-infected cells co-cultured with T cells promoted the differentiation of Treg cells. After 48 hpi, the levels of TGF-β mRNA (**A**) and protein (**B**) were detected. (**C**) Co-culture model with T cells. (**D**) The frequency of Treg cells. T cells were co-cultured for three days with PCV2-infected host cells (with inhibitors added as required). The quantity of Treg cells was determined under different treatments. (**E**) The MFI of Foxp3 in CD4^+^Foxp3^+^ double-positive cells. Foxp3 expression in Treg cells was quantified by calculating the arithmetic MFI using flow cytometry.

Due to the strong tissue tropism of PCV2, we infected three distinct cell lines (IPEC-J2, 3D4/21, and PK-15) *in vitro* and confirmed viral replication in all three cell types (Fig. S8). To identify potential sources of TGF-β, we measured TGF-β expression levels following PCV2 infection in these cell lines. And the results of qPCR and ELISA both indicated that PCV2 infection of three cell types could enhance the secretion of TGF-β (Fig. 6A and B). Furthermore, the PCV2-infected cells were co-cultured with T cells (Fig. 6C), and the differentiation of Treg cells was examined. The research discovered that the co-culture could induce the differentiation of Treg cells, while the addition of SIS3 or SB-431542 could reverse the outcome (Fig. 6D and E). Our study confirmed that PCV2 infection promoted the differentiation of Treg cells through the TGF-β/Smad3 pathway.

## DISCUSSION

PCV2 is a kind of immunosuppressive disease that is harmful to the pig industry. The destruction of lymphoid organ tissue structure and lymphocyte depletion are two crucial factors contributing to the persistent infection caused by PCV2. In order to survive, viruses employ a variety of mechanisms to inhibit or escape the host’s antiviral defenses. Above all, PCV2 has the ability to inhibit the function of immune cells. Studies have shown that PCV2 can disrupt the function of antigen-presenting cells (APCs), such as DCs, including the disrupting actin polymerization, interfering with skeletal rearrangement, reducing endocytosis of viral particles, and impacting antigen-presenting function (27–29). Secondly, PCV2 infection can regulate host cytokine expression and signal transduction. The recent study has shown that PCV2 can inhibit the activation of the cGAS-STING signaling pathway to block the production of type I interferon IFN-β, thus promoting PCV2 infection (30). And PCV2 Rep protein has an immunomodulatory CpG motif, which can inhibit IFN-α production (31). Besides, diseased pigs with PMWS can secrete IL-10, promote the secretion of inflammatory cytokines IL-1β and IL-8 (32), and inhibit the secretion of IL-2 and IL-4, so as to reduce the proliferation, differentiation, and antiviral effects of immune cells (33, 34). What is more, PCV2 can hide in various immune cells. Although PCV2 cannot proliferate effectively in DCs and mononuclear/macrophage cell lines, it has not lost its infectivity. DCs infected with PCV2 do not present effective information to T cells. Instead, they circulate in the body together with the host’s DCs for the purpose of dissemination. This indicates an immune cell-dependent evasion strategy for the virus (35). Furthermore, recent studies by the Nauwynck team have indicated that PCV2 can replicate in T cells (36). Based on the existing studies, we contend that both the efficient replication of the virus in lymphocytes and the inefficient clearance of it in monocytes are crucial reasons for the virus to evade the host’s immune response.

Previous studies have shown that PCV2 infection can decrease the frequency of T cell subsets including CD4^+^ T cells, CD3^+^CD4^+^CD8^+^ memory Th cells, CD3^+^CD4^+^CD8^−^ initial Th cells, CD3^+^CD4^−^CD8^+^ Tc cells, and CD3^+^CD4^−^CD8^−^γδTCR^+^ cells. However, the host immune balance of Th1/Th2/Th17/Treg has not been reported during the viral infection (37). Different subtypes of CD4^+^ T cells perform distinct functions: Th1 cells mainly secrete IL-2 and IFN-γ to engage in cellular immune responses, Th2 cells predominantly secrete IL-4 to participate in humoral immunity, and Th17 cells chiefly secrete Th17 to induce inflammation. Treg cells primarily secrete TGF-β and IL-10 to suppress the inflammatory response (38–40). Therefore, this study investigated the impact of PCV2 infection on the immune balance of CD4^+^ T cell subsets (Th1/Th2/Th17/Treg) to elucidate the molecular mechanisms underlying PCV2-induced immunosuppression. The results showed that PCV2-infected piglets exhibited a reduction in CD4^+^ T cell numbers at early stages, along with disrupted Th1/Th2/Th17/Treg balance and a shift toward Treg cell dominance. Furthermore, given the high tropism of PCV2 for the spleen and lymph nodes and its ability to induce Treg cell differentiation, we examined Foxp3 expression levels in these tissues. The results demonstrated significantly elevated Foxp3 expression in the spleen of PCV2-infected piglets, while lymph nodes showed an increasing trend that did not reach statistical significance (Fig. S4). We propose that this tissue-specific discrepancy may stem from cellular heterogeneity. Although T cells constitute the predominant population (60% ~ 70%) in both spleen and lymph nodes, the remaining 30% ~ 40% of cells may also contribute to Foxp3 expression. This is supported by our previous findings showing downregulated Foxp3 expression in PCV2-infected IPEC-J2, 3D4/21, and PK-15 cell lines (41). Thus, qPCR or Western blot data represent the aggregate Foxp3 expression from diverse cell types within tissues. For a comprehensive investigation of T cell differentiation, integrated analysis of mRNA, protein levels, and flow cytometry data is warranted.

Among the different subtypes of CD4^+^ T cells, Treg cells possess unique immune inhibition properties, which enable them to serve a dual role. On the one hand, Treg cells benefit the host by suppressing excessive inflammation that can be harmful to the host tissue, but on the other hand, they can promote the replication and persistence of pathogens by limiting potentially protective immune responses (42, 43). Recent studies by Li’s research team have demonstrated that PCV2-infected IPEC-J2 cells activated the NF-κB signaling pathway to promote TGF-β synthesis and subsequently induced Treg cell differentiation through ERK activation (22), with viral ORF4 protein identified as the key effector regulating TGF-β secretion (23). While these findings provide novel insights, downstream mechanisms specifically governing Treg cell differentiation remain unclear. To elucidate the clinical relevance of PCV2-induced Treg cell differentiation, we first systematically evaluate the virus’s impact on host immune homeostasis, with particular focus on the Th1/Th2/Th17/Treg cell balance. This comprehensive immunological profiling will provide the necessary context for mechanistic investigations into Treg cell differentiation. In addition, current research lacks direct evidence of TGF-β secretion during natural PCV2 infection, necessitating clinical sample analysis to establish *in vivo-in vitro* correlation. Importantly, studies should expand beyond one cell line to other susceptible cell lines and animal models to verify pathway universality. During our *in vitro* studies, we attempted to validate our findings using primary porcine alveolar macrophages (PAMs). However, PCV2 infection of PAMs followed by co-culture with T cells failed to induce Treg cell differentiation. Experimental data demonstrated that PCV2 infection of PAMs did not result in significant viral proliferation (Fig. S9A), nor was the expression of viral Cap detected (Fig. S9B). Further qPCR and ELISA analyses revealed no notable changes in TGF-β mRNA expression levels post-PCV2 infection (Fig. S9C), while the protein secretion levels exhibited a decreasing trend (Fig. S9D). These findings suggest that PAMs may not serve as an optimal *in vitro* model system for PCV2 research.

Apart from the fact that cytokines can affect the differentiation of T cells, an increasing number of studies have indicated that the gut microbiota is also involved in the differentiation process of T cells (44, 45). Notably, several species of intestinal bacteria, such as *Lactobacillus*, have been associated with enhanced efficacy of checkpoint blockade immunotherapy (46). Furthermore, a recent study has shown that *Collinella aerofaciens* has the ability to produce butyrate (47). While studying the immune balance of CD4^+^ T cells, we found that PCV2 infection can cause changes in the intestinal microbiota of piglets (Fig. S10). In our research, we also noticed a significant increase of *Collinella aerofaciens* and another butyrate-producing bacteria, *Gemmiger formicilis,* in the challenge group. These bacteria have been known to promote short-chain fatty acids (SCFAs) secretion (48), which can induce Treg cell differentiation and impair inflammation suppression (49, 50). Additionally, we observed that *A. muciniphila*, a bacteria related to a healthy intestinal barrier and immune balance, was significantly reduced in the challenge group. The deficiency of *A. muciniphila* is an important factor causing inflammation (51, 52). This may be the cause of diarrhea in the challenge piglets, and it also reflects the dynamic change process of the host in order to maintain homeostasis.

**Fig 7:**
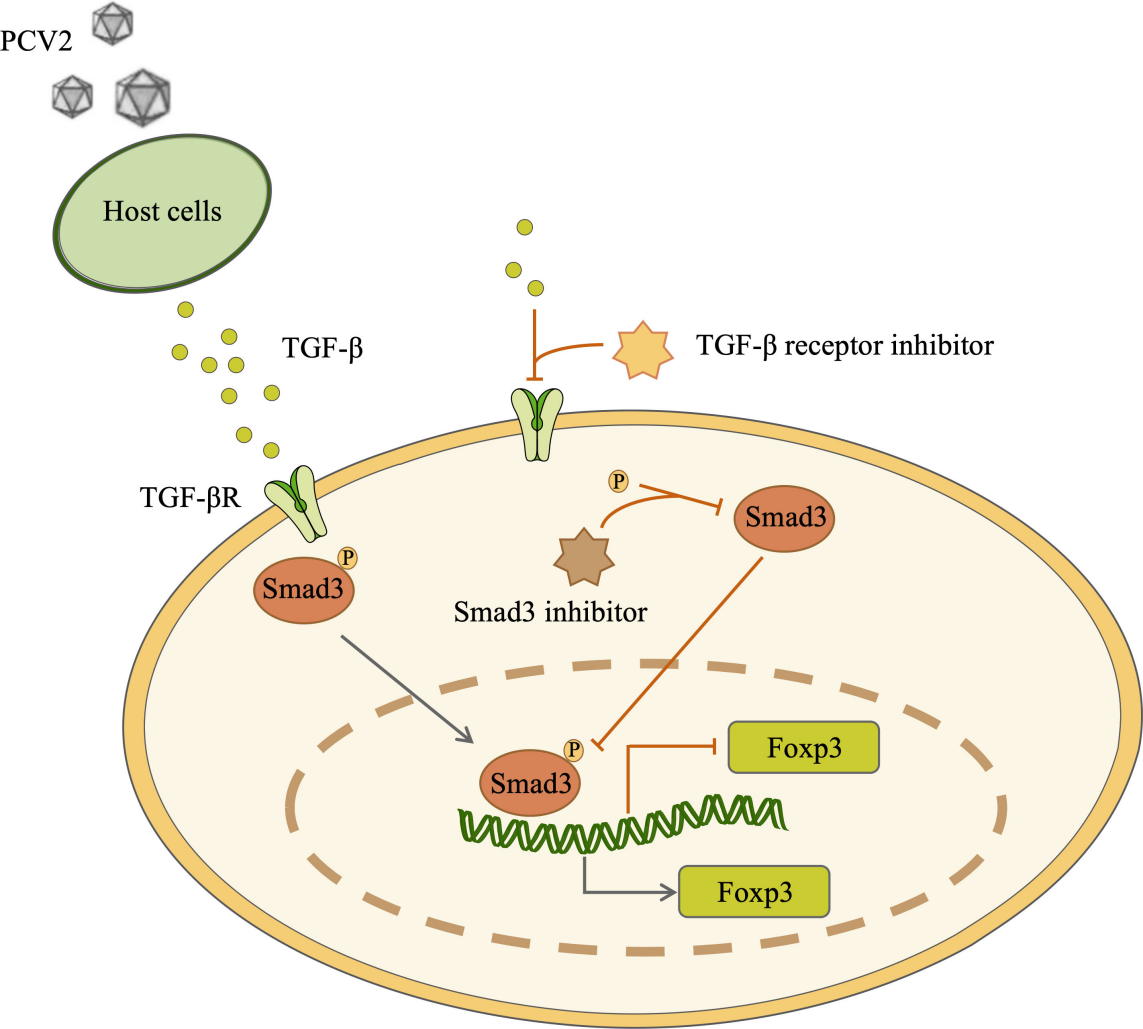
The schematic diagram indicates that PCV2 infection induces the differentiation of Treg cells via the TGF-β/Smad3 pathway. PCV2 infection markedly enhances TGF-β secretion from host cells, subsequently activating the Smad3 signaling pathway in T cells. Phosphorylated Smad3 translocates into the nucleus, specifically binds to the Foxp3 gene enhancer region, and significantly increases Foxp3 transcription, ultimately promoting Treg cell differentiation. Notably, treatment with the Smad3-specific inhibitor SIS3 and TGF-β receptor inhibitor SB-431542 effectively inhibits Treg differentiation, confirming the crucial role of this pathway in PCV2-induced Treg cell differentiation.

In summary, PCV2 can facilitate the secretion of TGF-β. After being stimulated by the cytokine, the cells recruit Smad3 and phosphorylate it. Smad3 further conveys the signal to the nucleus and binds to the enhancer of Foxp3, thereby enhancing the transcription level of Foxp3 and facilitating the differentiation of Treg cells (Fig. 7). In subsequent studies, we hold that it is requisite to further determine whether PCV2 affects Foxp3 post-translational modifications (e.g., acetylation, phosphorylation, ubiquitination) (53–55). Because this is not only conducive to the stability of Foxp3, but also to the functional exertion of Treg cells. Perhaps this is one of the approaches by which PCV2 infection frequently presents as a latent form and induces immune suppression.

## SUPPLEMENTAL MATERIAL

Supplemental material mbio.01366-25-s0001.docx Fig. S1 to S10; Tables S1 and S2.

ASM does not own the copyrights to Supplemental Material that may be linked to, or accessed through, an article. The authors have granted ASM a non-exclusive, world-wide license to publish the Supplemental Material files. Please contact the corresponding author directly for reuse.
