# Supplementary material for: PCV2 infection induces the differentiation of Treg cells via the TGF-β/Smad3 pathway
Source: mBio. 2025 Jul 31;16(9):e01366-25. doi: 10.1128/mbio.01366-25 (PMC12421831; doi:10.1128/mbio.01366-25)
Supplement: Supplemental material — Fig. S1 to S10; Tables S1 and S2. [file mbio.01366-25-s0001.docx]

**SUPPLEMENTAL TABLE**

Supplemental table 1 Clinical Scoring Criteria

| Category | Detail description | Score |
| --- | --- | --- |
| Temperature | T ≤39.9 | 0 |
|  | 40.0≤T≤40.9 | 1 |
|  | 41.0≤T | 2 |
| Digestive tract symptom | Normal | 0 |
|  | Mild diarrhea (pasty soft stools) | 1 |
|  | Severe diarrhea (unformed loose stools) | 2 |
|  | Inappetence | 1 |
|  | Anorexia/Fasting | 2 |
| Mental status | Normal | 0 |
|  | Mild lethargy | 1 |
|  | Severe lethargy | 2 |
| Respiratory status | Normal | 0 |
|  | Tachypnea | 1 |
|  | Dyspnea | 2 |

Supplemental table 2 qPCR primers information

| Name | Primer sequences (5’-3’) |
| --- | --- |
| PCV2-pET-28a-F | taagaaggagatataccatggGGCCAGTTCGTCACCCTTTC |
| PCV2-pET-28a-R | gtggtggtggtggtgctcgagCAGTATATACGACCAGGAATACAATATCC |
| PCV2-qPCR-F | TGCCAGTTCGTCACCCTTT |
| PCV2-qPCR-R | CAGTATATACGACCAGGACTACAATATC |
| T-bet-qPCR-F | ACAGCTACGAGGCCGAATTT |
| T-bet-qPCR-R | CATCTTGGGAGGGTACTGCG |
| GATA3-qPCR-F | GTGCAAAAAGGTGCACGACA |
| GATA3-qPCR-R | CCATACTGGAAGGGTGGTGG |
| RORγ-qPCR-F | TTTTGAGGACCCCAGGCATC |
| RORγ-qPCR-R | TCTCTGTCAGGGAGGCGTAA |
| Foxp3-qPCR-F | CCTTTCACATACGCCACCCT |
| Foxp3-qPCR-R | CTGAAGAAGGCGAACATGCG |
| β-actin-qPCR-F | CTCCATCATGAAGTGCGACGT |
| β-actin-qPCR-R | GTGATCTCCTTCTGCATCCTGTC |

**SUPPLEMENTAL FIGURES**


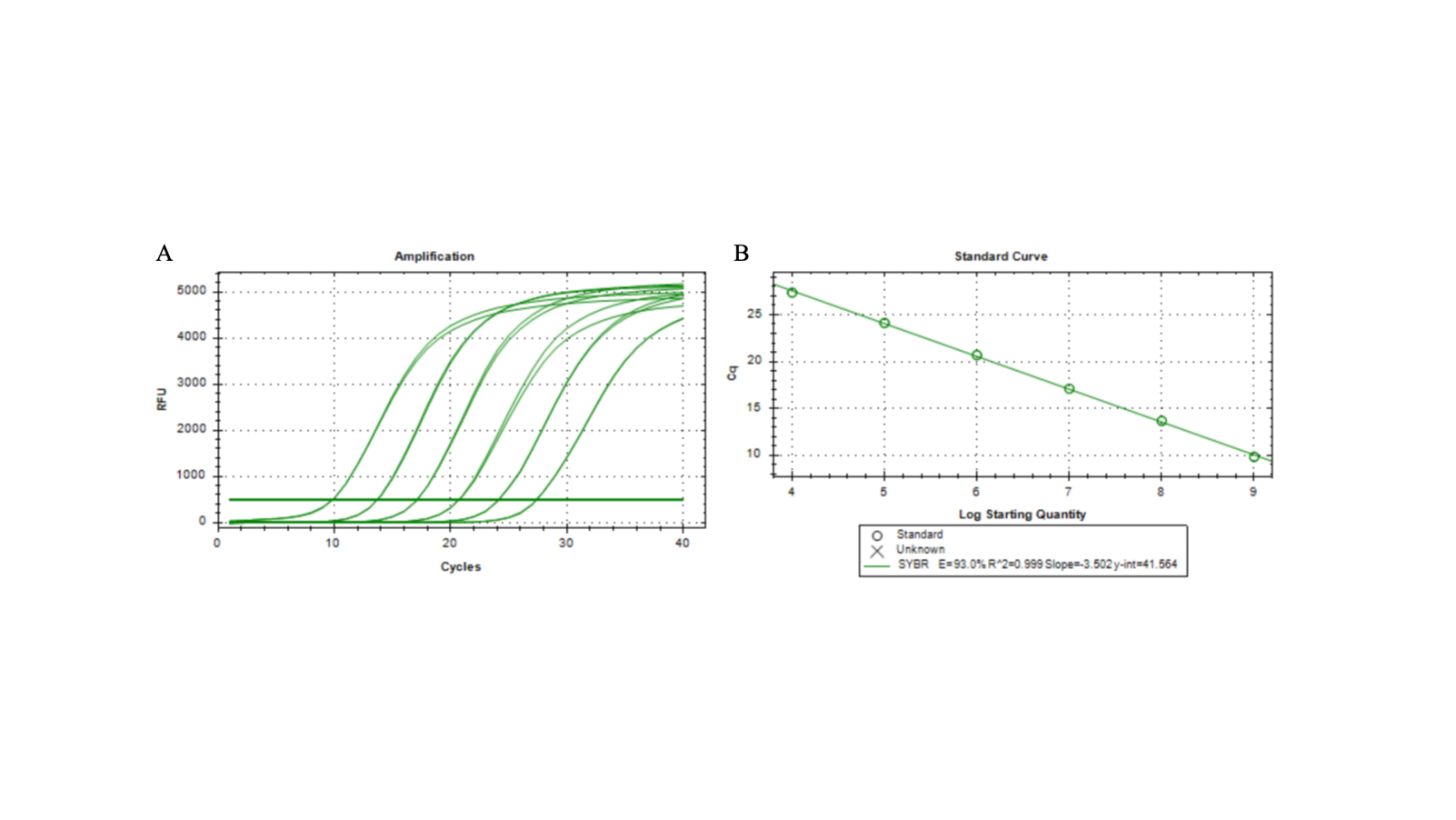


**Supplemental Fig. 1: Establishment of PCV2 qPCR detection method.** (A) Amplification curve. The pET-28a-Rep plasmid was quantified to 1×10^10^ copies/μL, followed by a 10-fold gradient dilution as a DNA template. (B) Standard curve. According to the relationship between virus copy number and Ct value, the final formula was obtained: Y=-3.502(lgX)+41.564.


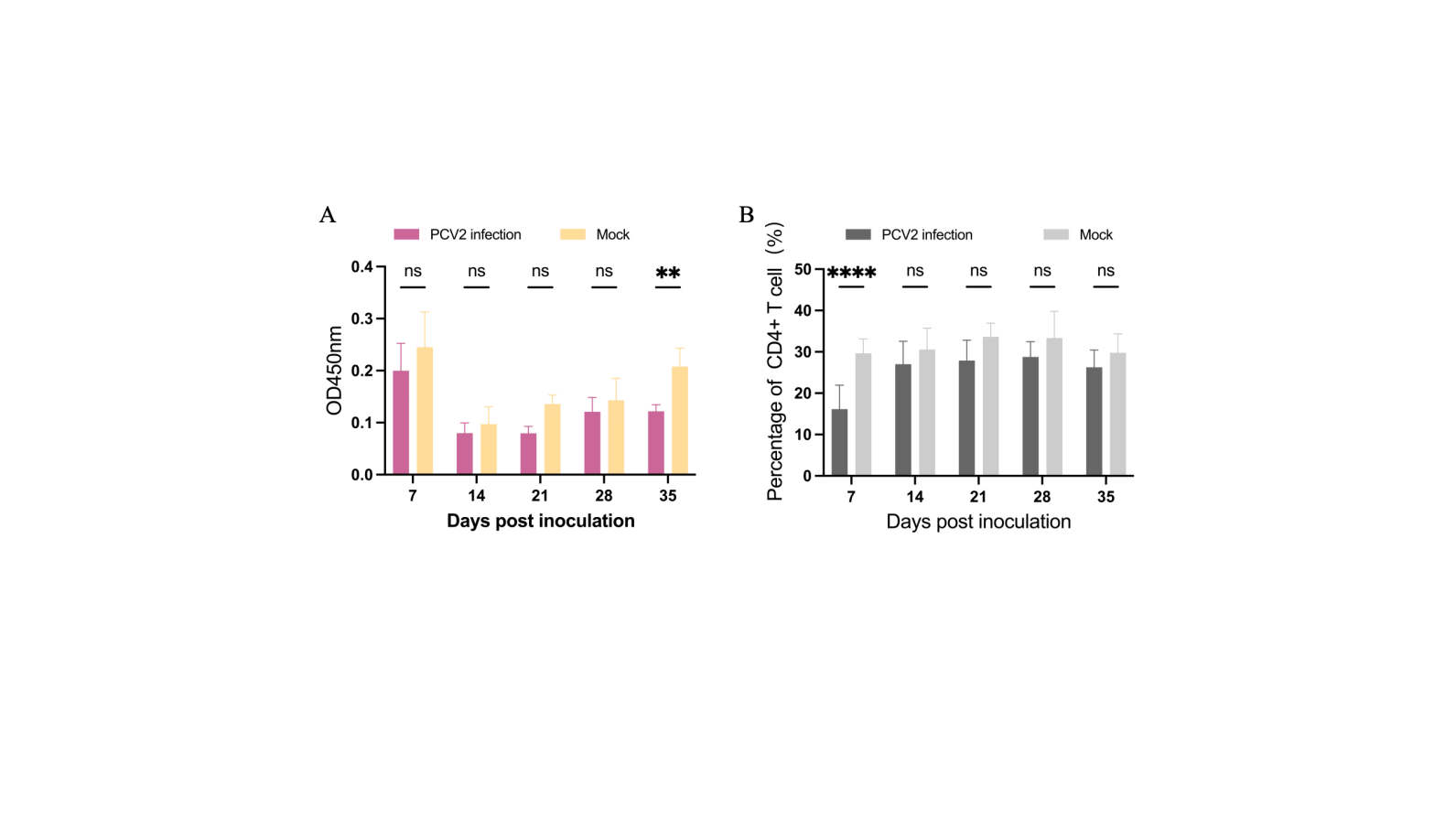


**Supplemental Fig. 2: Analysis of T cells proliferation capacity and CD4^+^ T cells population dynamics.** (A) Detection of T cells proliferation ability. T cells isolated from PBMCs at different post-challenge intervals were stimulated *in vitro* for 72 h, with proliferation measured via MTT assay. (B) PBMCs were isolated at various time points post-challenge, and CD4^+^ T cells populations were quantified by flow cytometry.


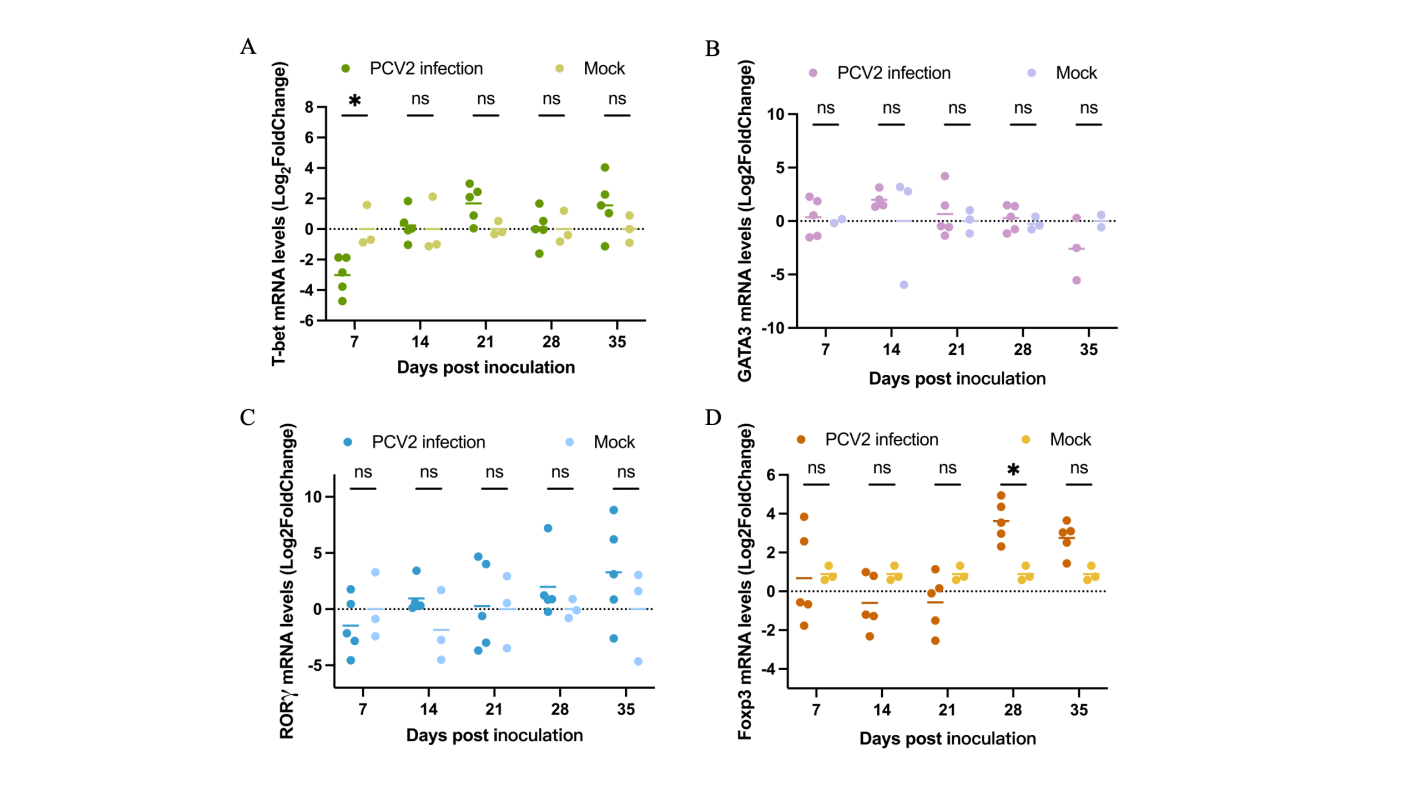


**Supplemental Fig. 3: T cells transcription factor detection.** (A) T-bet mRNA levels. (B) GATA3 mRNA levels. (C) RORγ mRNA levels. (D) Foxp3 mRNA levels. PBMCs were isolated from peripheral blood at multiple time points post-challenge, and T cells specific transcription factors were quantified by qPCR. In terms of details, T-bet, GATA3, RORγ, and Foxp3 are the characteristic transcription factors of Th1, Th2, Th17, and Treg, respectively. These transcription factors can be used to indicate the frequency of different CD4^+^ T cells.


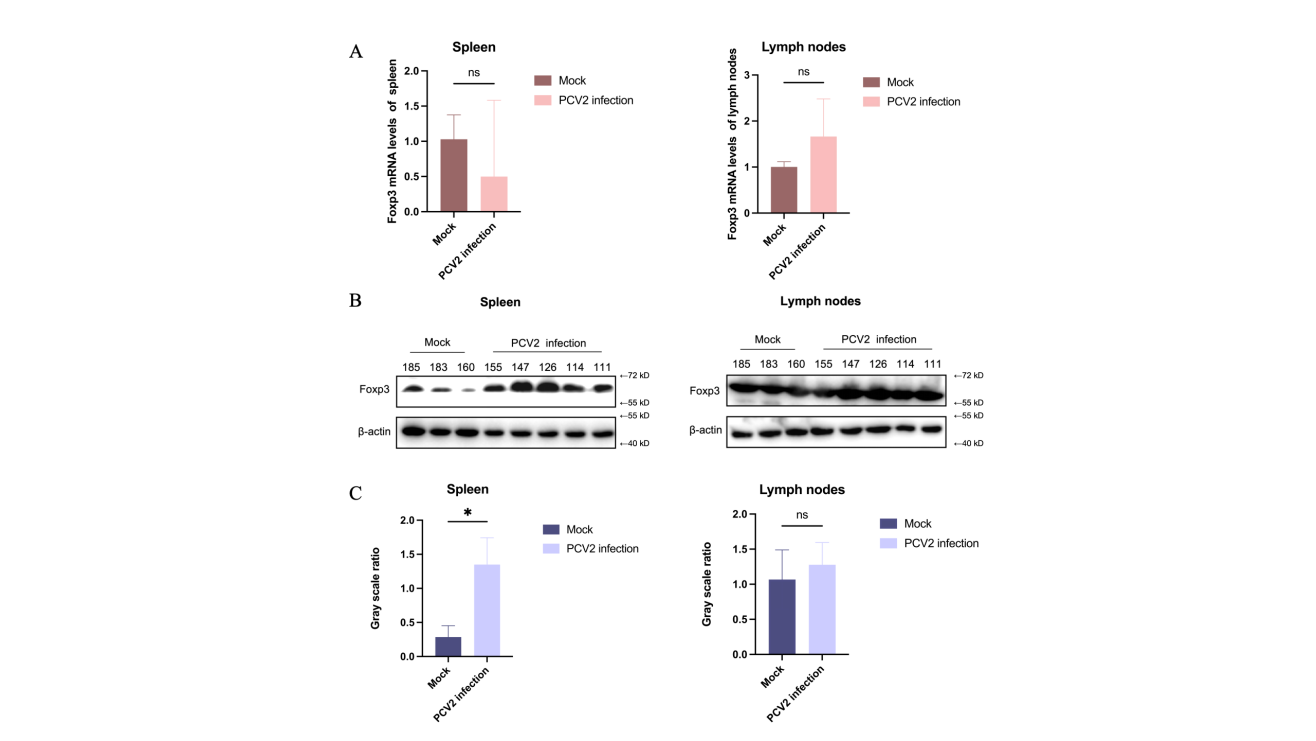


**Supplemental Fig. 4: Analysis of Foxp3 mRNA and protein expression levels.** (A) Quantification of Foxp3 mRNA levels in the spleen and lymph nodes. At 35 dpi, piglets were euthanized, and tissue samples were collected for RNA extraction followed by qPCR analysis. (B) Detection of Foxp3 protein levels in the spleen and lymph nodes. Porcine tissue proteins were extracted and subjected to Western blot analysis to determine Foxp3 protein expression levels. (C) Gray-scale analysis of the protein bands in (B).


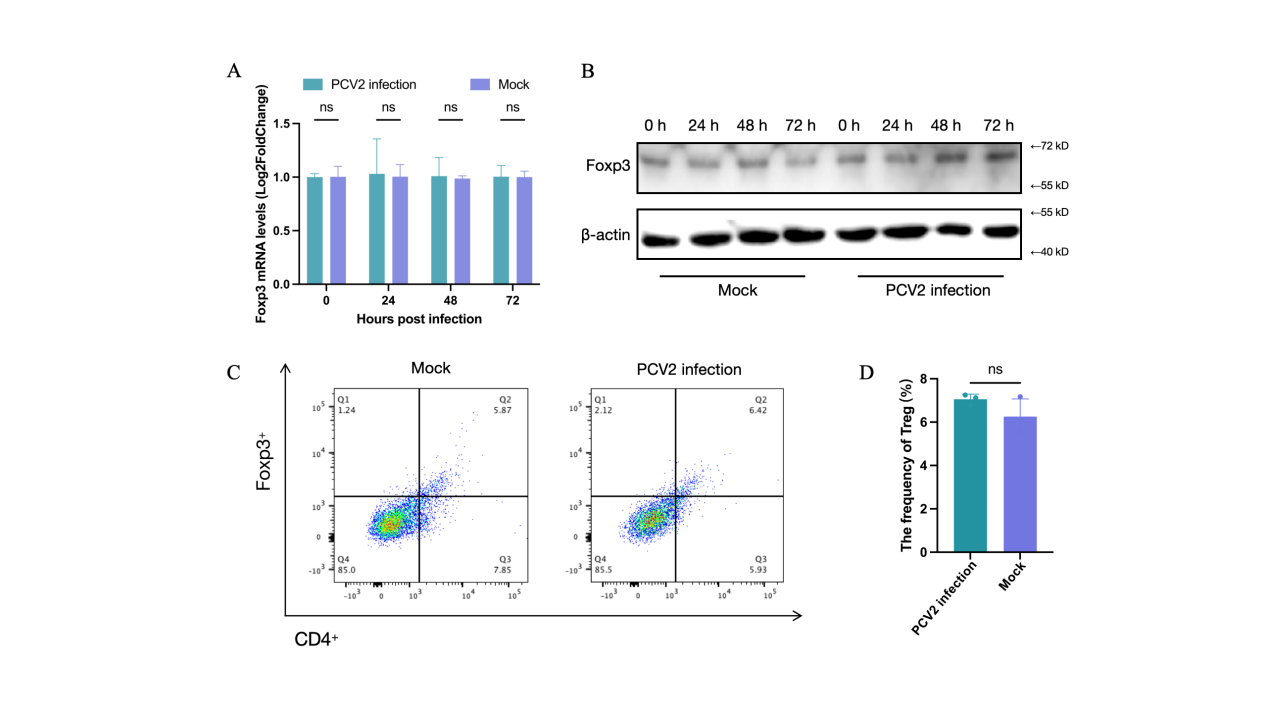


**Supplemental Fig. 5: Impact of direct PCV2 infection on Treg cells differentiation.** (A) Quantification of Foxp3 mRNA levels in T cells. (B) Foxp3 expression profiling in T cells. (C) Flow cytometric detection of Treg cells. T cells were infected with PCV2 *in vitro* for 72 h, followed by flow cytometric analysis to quantify Treg cells populations. (D) Statistical results of Treg cells proportions.


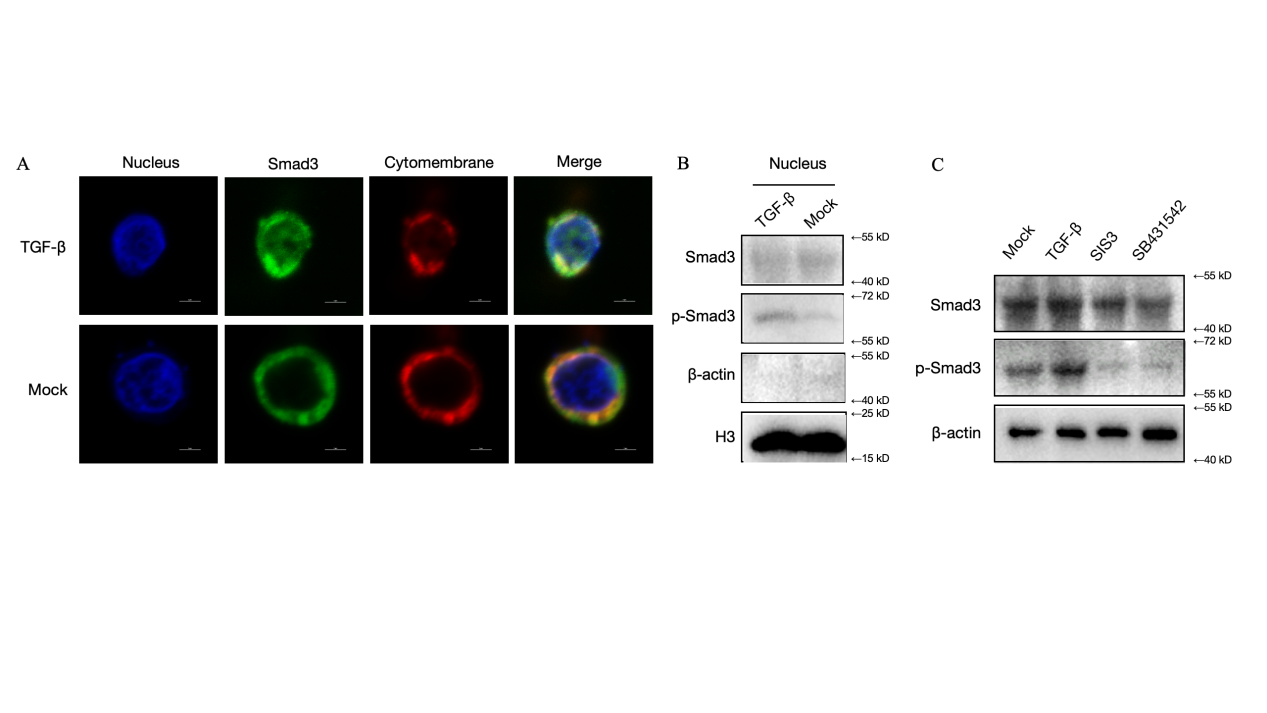


**Supplemental Fig. 6: Subcellular localization and phosphorylation analysis of Smad3.** (A) Confocal microscopy analysis of Smad3 subcellular localization. Following 5 days TGF-β stimulation of cultured T cells, plasma membranes were stained using CellMask™ Orange Plasma Membrane Stain kit (C10045, Invitrogen™), and the localization of Smad3 was examined by laser confocal microscopy. (B) Analysis of phosphorylated Smad3 in nuclear compartments. After 5 days of TGF-β stimulation in cultured T cells, nuclear proteins were extracted and Smad3 phosphorylation levels were analyzed by Western blot. (C) The effect of inhibitor treatment on Smad3 phosphorylation. Following 5 days inhibitor treatment of T cells, total proteins were extracted for simultaneous detection of Smad3 expression and its phosphorylation.


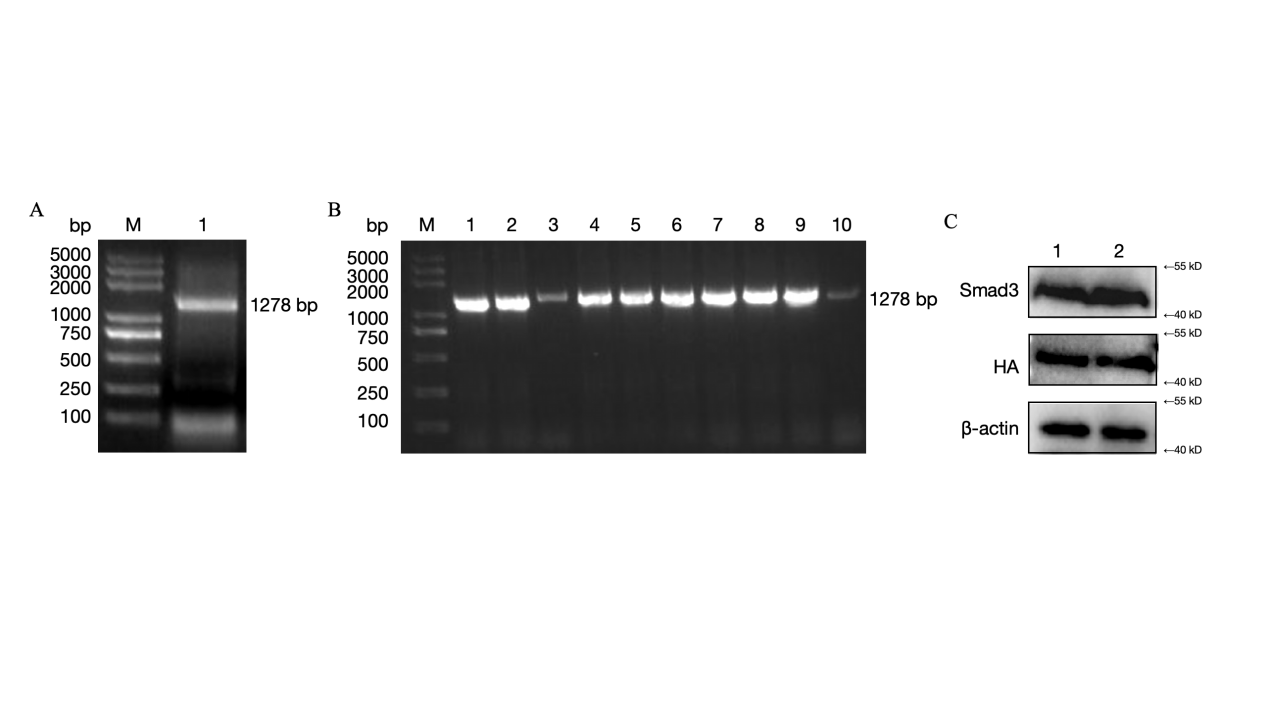


**Supplemental Fig. 7: Construction of Smad3 eukaryotic expression plasmid.** (A) Amplification of the *Smad3* gene. (B) Smad3 was ligated to the pCAGGS-HA plasmid and transformed into DH5ɑ, and single clones were picked for identification. (C) Two successfully constructed plasmids, No. 1 and No. 2, were selected. After transfection into HEK-293T cells, the expression of Smad3 protein was determined by Western blot.


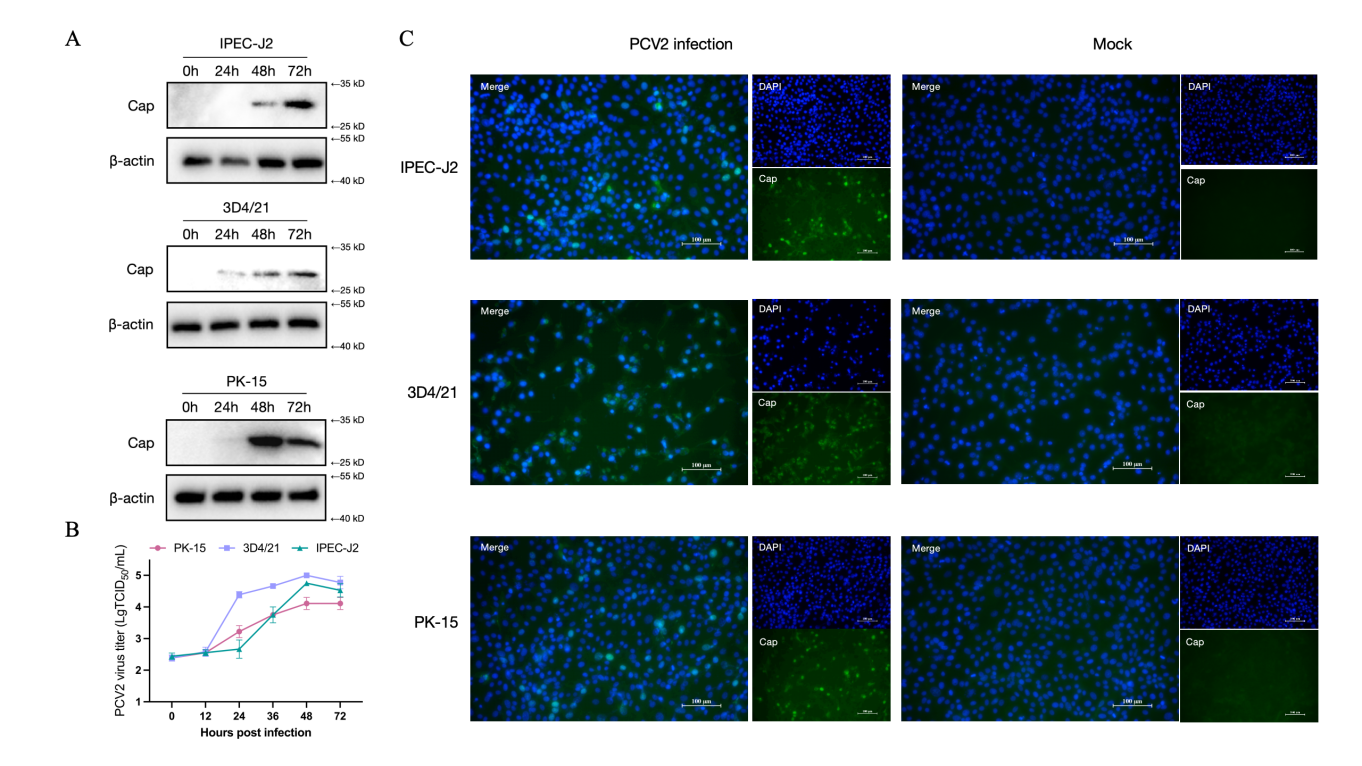


**Supplemental Fig. 8: Propagation characteristics of PCV2 in different cell lines.** (A) Detection of Cap by Western blot. Following PCV2 infection of IPEC-J2, 3D4/21, and PK-15 cell lines, total cellular proteins were collected at various time points and analyzed for Cap expression by Western blot. (B) Replication kinetics of PCV2 in three host cell lines. Viral titers of PCV2 at different time points were determined by TCID_50_ assay. (C) Detection of Cap by IFA. PCV2-infected cells were analyzed by IFA at 48 hpi to assess Cap expression and localization.


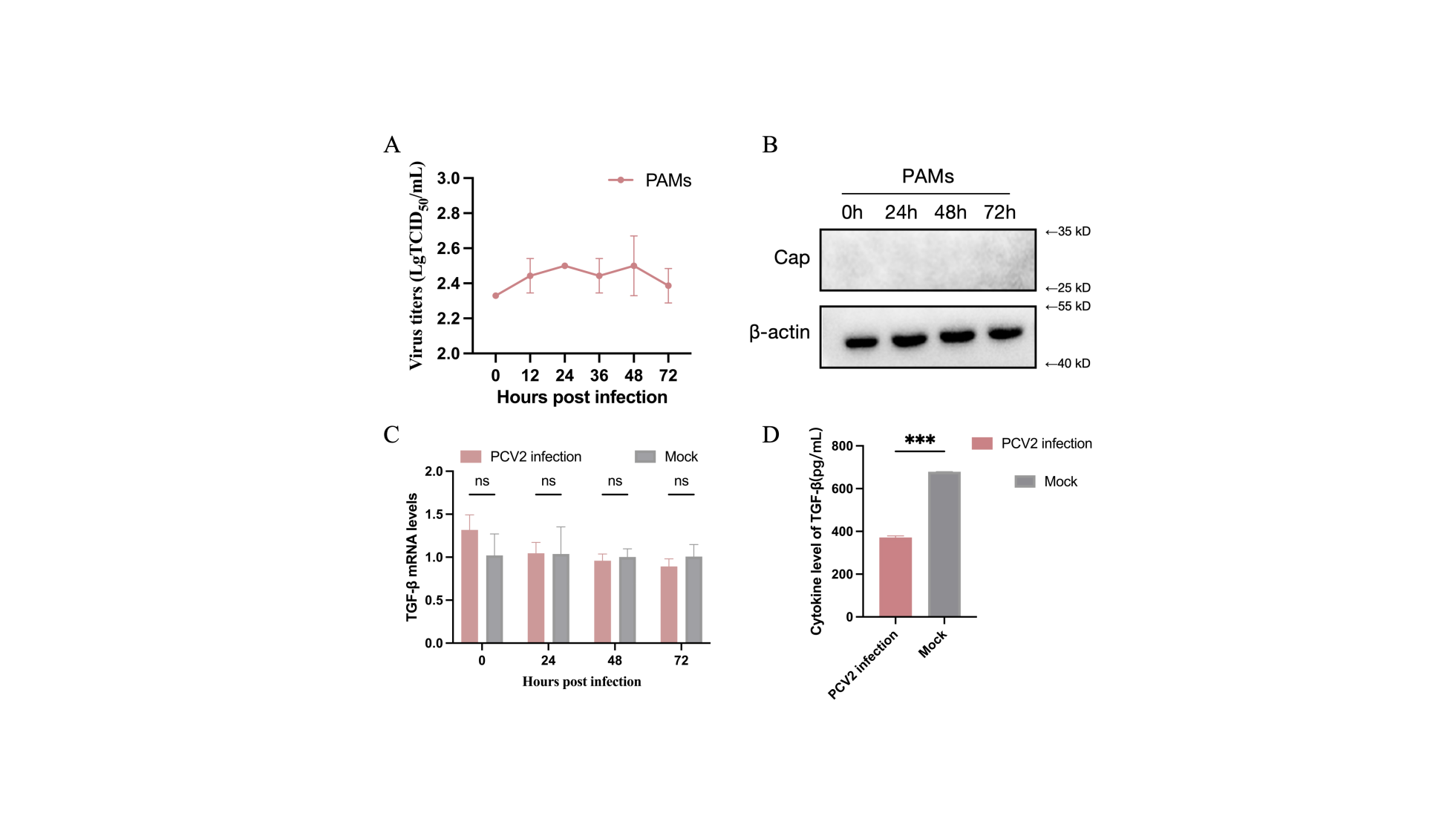


**Supplemental Fig. 9: PCV2 replication dynamics and TGF-β expression profiles in PAMs.** (A) PCV2 replication dynamics in PAMs. PCV2 replication in PAMs was assessed following infection at MOI=0.1. Viral titers in culture supernatants collected at indicated time points were determined by TCID_50_ assay. (B) PCV2 Cap expression was examined by Western blot in PAMs (MOI=0.1), with total proteins extracted at designated time points. (C) TGF-β mRNA levels. PCV2-infected PAMs (MOI=0.1) were assessed by qPCR at indicated time points. (D) TGF-β cytokine analysis. TGF-β secretion was evaluated by ELISA in 48 hpi culture supernatants.


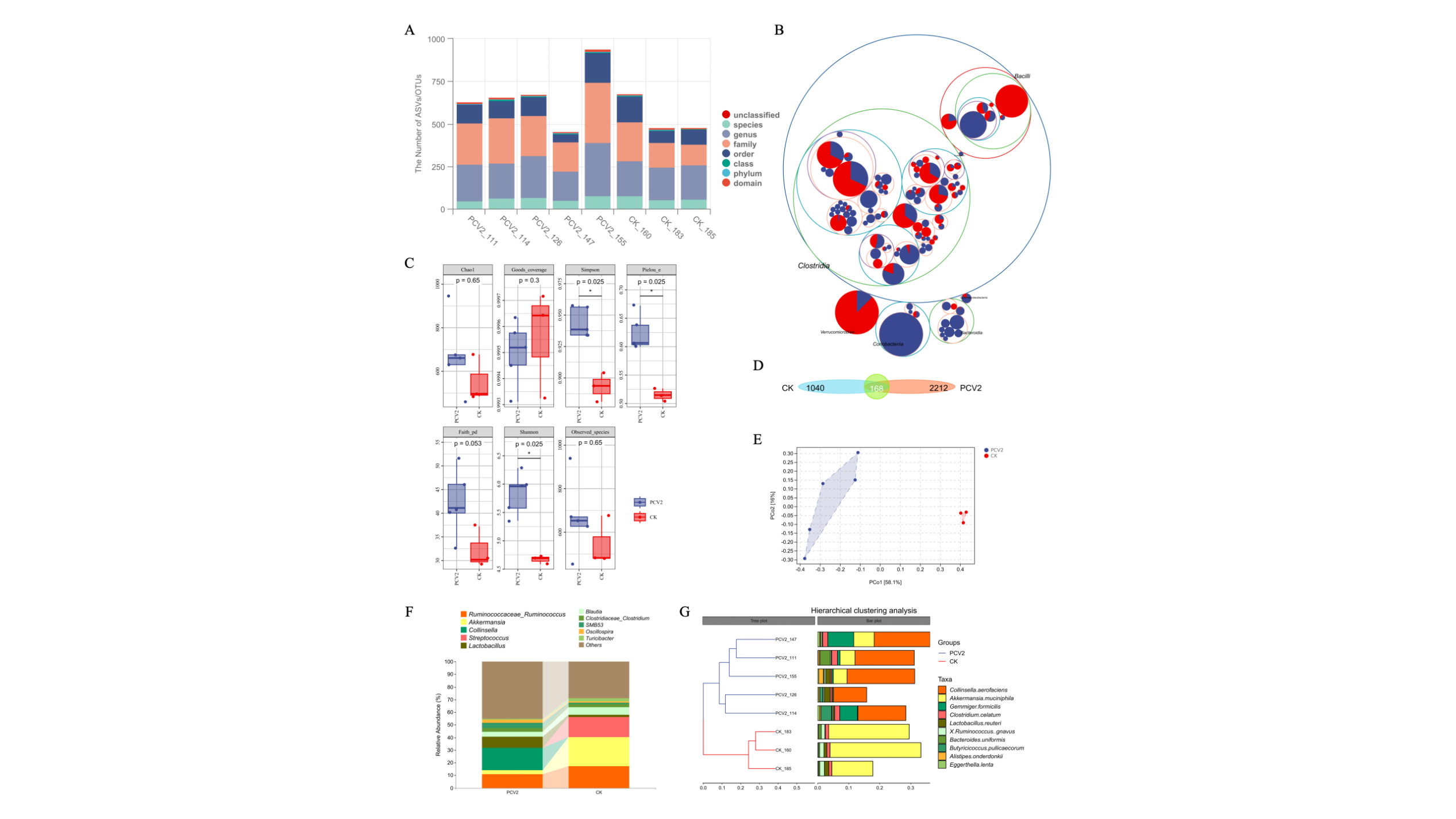


**Supplemental Fig. 10: Comparative analysis of the gut microbial community structure in different groups.** (A) The quantity of ASVs in different piglet samples. (B) Taxonomic notes. (C) Analysis of alpha diversity. (D) Venn diagram of comparison of ASV distribution in different groups. (E) Principal coordinate analysis. (F) Relative abundance of the gut microbiota in different groups. (G) Visualization of different species in each sample. The panel on the left is a hierarchical clustering tree, where samples are grouped according to their similarity. The shorter the branch length between samples, the more similar the two samples are. The panel on the right (drawn by default) is a stacked bar chart of the top 10 species in abundance.
